# The complete mitochondrial DNA of three monozoic tapeworms in the Caryophyllidea: a mitogenomic perspective on the phylogeny of eucestodes

**DOI:** 10.1186/s13071-017-2245-y

**Published:** 2017-06-27

**Authors:** Wen X. Li, Dong Zhang, Kellyanne Boyce, Bing W. Xi, Hong Zou, Shan G. Wu, Ming Li, Gui T. Wang

**Affiliations:** 1 Key Laboratory of Aquaculture Disease Control, Ministry of Agriculture, and State Key Laboratory of Freshwater Ecology and Biotechnology, Institute of Hydrobiology, Chinese Academy of Sciences, Wuhan, 430072 China; 2 University of Chinese Academy of Sciences, Beijing, 100049 China; 3 South Devon College University Centre, Long Road, Paignton, TQ4 7EJ UK; 4 Key Laboratory of Freshwater Fisheries and Germplasm Resources Utilization, Ministry of Agriculture, Freshwater Fisheries Research Center, Chinese Academy of Fishery Sciences, Wuxi, 214081 China

**Keywords:** Mitogenome, Caryophyllidean tapeworm, Parasitic Platyhelminthes, Proglottization, Segmentation

## Abstract

**Background:**

External segmentation and internal proglottization are important evolutionary characters of the Eucestoda. The monozoic caryophyllideans are considered the earliest diverging eucestodes based on partial mitochondrial genes and nuclear rDNA sequences, yet, there are currently no complete mitogenomes available. We have therefore sequenced the complete mitogenomes of three caryophyllideans, as well as the polyzoic *Schyzocotyle acheilognathi*, explored the phylogenetic relationships of eucestodes and compared the gene arrangements between unsegmented and segmented cestodes.

**Results:**

The circular mitogenome of *Atractolytocestus huronensis* was 15,130 bp, the longest sequence of all the available cestodes, 14,620 bp for *Khawia sinensis*, 14,011 bp for *Breviscolex orientalis* and 14,046 bp for *Schyzocotyle acheilognathi*. The A-T content of the three caryophyllideans was found to be lower than any other published mitogenome. Highly repetitive regions were detected among the non-coding regions (NCRs) of the four cestode species. The evolutionary relationship determined between the five orders (Caryophyllidea, Diphyllobothriidea, Bothriocephalidea, Proteocephalidea and Cyclophyllidea) is consistent with that expected from morphology and the large fragments of mtDNA when reconstructed using all 36 genes. Examination of the 54 mitogenomes from these five orders, revealed a unique arrangement for each order except for the Cyclophyllidea which had two types that were identical to that of the Diphyllobothriidea and the Proteocephalidea. When comparing gene order between the unsegmented and segmented cestodes, the segmented cestodes were found to have the lower similarities due to a long distance transposition event. All rearrangement events between the four arrangement categories took place at the junction of *rrnS*-*tRNA* ^*Arg*^ (P1) where NCRs are common.

**Conclusions:**

Highly repetitive regions are detected among NCRs of the four cestode species. A long distance transposition event is inferred between the unsegmented and segmented cestodes. Gene arrangements of Taeniidae and the rest of the families in the Cyclophyllidea are found be identical to those of the sister order Proteocephalidea and the relatively basal order Diphyllobothriidea, respectively.

**Electronic supplementary material:**

The online version of this article (doi:10.1186/s13071-017-2245-y) contains supplementary material, which is available to authorized users.

## Background

Scolex type, external segmentation and internal proglottization are all important evolutionary characters of the Cestoda. The Amphilinidea and Gyrocotylidea (Cestodaria) that do not possess a scolex are early divergent lineages in this class. Tapeworms of the order Caryophyllidea (Platyhelminthes: Eucestoda) are typified by a monozoic body (neither internal proglottization nor external segmentation). The Spathebothriidea are polyzoic but externally unsegmented, and all other eucestodes demonstrate classic proglottization (segmented body parts each with a set of reproductive organs). Morphological analysis shows the Caryophyllidea to be the earliest divergent lineage of Eucestoda [1] although phylogenetic analysis based on LSU rDNA and SSU rDNA have indicated that the Spathebothriidea may be the earliest diverging eucestodes [2, 3]. However, recently, topology constructed using large fragments of mtDNA supports the Caryophyllidea as the most primitive eucestodes [4]. These results indicate the Caryophyllidea to be a key group for studying evolutionary relationships within the Eucestoda as well as with other parasitic Monogenea, Aspidogastrea and Digenea.

Owing to its maternal inheritance, a lack of recombination and a fast rate of evolution [5], the haploid mitochondrial genome has proven to be a useful marker for population studies, species identification and phylogenetics [6, 7]. Its genome-level characteristics, gene arrangements and the positions of mobile genetic elements also enable it to be a powerful tool for reconstructing evolutionary relationships [8–10]. Using gene sequences and gene arrangements from the complete mt genome, the phylogenies of some parasitic Platyhelminthes have been reconstructed [11–13]. However, due to a paucity of complete mt genomic information from these groups, very few parasitic flatworms have been included in these phylogenetic analyses. From the 16 orders of cestodes that exist, only four (Diphyllobothriidea, Bothriocephalidea, Proteocephalidea and Cyclophyllidea) are currently represented in the GenBank database, and as the ancestral taxa of the Eucestoda, no complete mitogenome from the Caryophyllidea has been sequenced.

*Khawia sinensis* Hsü, 1935, and *Atractolytocestus huronensis* Anthony, 1958, belong to the family Lytocestidae and are very common caryophyllideans in the intestine of the common carp (*Cyprinus carpio*). Both invasive tapeworms have a worldwide distribution and are translocated with the introduction of the common carp into countries around the world [14, 15]. *Breviscolex orientalis* Kulakovskaya, 1962, the only member of the family Capingentidae, is typically recorded in the cyprinids *Hemibarbus barbus* [16]. In addition, the Asian fish tapeworm *Schyzocotyle acheilognathi* (syn. *Bothriocephalus acheilognathi*), a segmented tapeworm of the Bothriocephallidea, is also an invasive parasite found worldwide.

This study has therefore generated the complete mitogenomes of three caryophyllideans, in addition to the Asian fish tapeworm in order to analyse the phylogenetic relationships of eucestodes and the differences in the gene arrangement between unsegmented and segmented eucestodes.

## Methods

### Specimen collection and DNA extraction

The following cestodes, *K. sinensis* and *A. huronensis* from the common carp (*Cyprinus carpio*), *B. orientalis* from *Hemibarbus maculates* and *S. acheilognathi* from the grass carp (*Ctenopharyngodon idella*), were collected from a fishery (29°59′10.47″N, 115°47′37″E) in Hubei Province, China. The parasites were preserved in 80% ethanol and stored at 4 °C. Specimens were stained with carmine and identified morphologically using the scolex and testis [16]. Total genomic DNA was extracted from the posterior region of a single tapeworm using a TIANamp Micro DNA Kit (Tiangen Biotech, Beijing, China), according to the manufacturer’s instructions. DNA was stored at -20 °C for subsequent molecular analysis. The morphological identification of specimens was verified by sequence analysis of the complete ITS1 rDNA region [17] and partial sequence of *cox*1 gene [18].

### PCR and DNA sequencing

Partial sequences of the mtDNA from the four cestodes were initially amplified by PCR using degenerate primers (Additional file 1: Table S1). Using these fragments, specific primers were designed for subsequent PCR amplification (Additional file 1: Table S1). PCR reactions were conducted in a 20 μl reaction mixture, containing 7.4 μl molecular grade water, 10 μl 2 × PCR buffer (Mg^2+^, dNTP plus, Takara, Dalian, China), 0.6 μl of each primer, 0.4 μl rTaq polymerase (250 U/μl, Takara), and 1 μl DNA template. Amplification was performed under the following conditions: initial denaturation at 98 °C for 2 min, followed by 40 cycles at 98 °C for 10 s, 48–60 °C for 15 s, 68 °C for 1 min/kb, and a final extension at 68 °C for 10 min. PCR products were sequenced bidirectionally at Sangon Company (Shanghai, China) using the primer walking strategy.

### Sequence analyses

The complete mt sequences were assembled manually and aligned against the mitogenome sequences of other published cestodes using the program MAFFT 7.149 [19] to determine the gene boundaries. Protein-coding genes (PCGs) were inferred with the help of BLASTX [20] and SeqBuilder module in the Lasergene7 software package (DNASTAR), employing the genetic code 9, the echinoderm and flatworm mitochondrial. The majority of tRNAs were identified by comparing the results of tRNAscan-SE [21], ARWEN [22], MITOs [23] and DOGMA [24]. However, *tRNA* ^*Phe*^ and *tRNA* ^*Gln*^ from *B. orientalis* and *tRNA* ^*Gln*^ from *A. huronensis* were visually compared with the sequences from other cestodes. The location of the two ribosomal RNA genes, *rrnL* and *rrnS,* were explored through alignment with other available mt cestodes sequences, and their ends were assumed to extend to the boundaries of their flanking genes. The 5′ end of the *rrnL* gene in *S. acheilognathi* however, was determined by the result of alignments. MitoTool [25], a home-made program, was primarily used to parse the annotated mt genome into a Word document format, and generate *.sqn file for GenBank submission and a *.csv file for Table 1. Mitotool was furthermore employed to unify the name of all 36 genes (12 PCGs, 2 rRNAs and 22 tRNAs) and locate all NCR positions (setting threshold of 50 bp) within the mitogenomes of the selected cestodes. Finally, the fasta file containing the nucleotide sequences and gene order for all 36 genes (12 PCGs, 2 rRNAs and 22 tRNAs) was extracted from the GenBank files, processed and used to generate Additional file 2: Table S2 and Additional file 3: Table S3. Repetitive regions within the NCRs were found using a local version of a Tandem Repeats Finder [26]. The alignments located in highly repetitive regions (HRRs) were shaded and labelled using TEXshade software [27]. The secondary structure of each consensus repeat unit was predicted by Mfold software [28], and codon usage and relative synonymous codon usage (RSCU) were computed with MEGA 5 [29]. CREx program [30] was then utilised to calculate the rearrangement events and to conduct pairwise comparisons of gene orders from all of the cestodes using common intervals measurement.

**Table 1:** The annotated mitochondrial genome of the four cestodes

Gene	Position		Size	Intergenic nucleotides	Codon		Anti-codon	Gene	Position		Size	Intergenic nucleotides	Codon		Anti-codon
	From	To			Start	Stop			From	To			Start	Stop	
(A) *Atractolytocestus huronensis*								(B) *Breviscolex orientalis*							
*cox*3	1	643	643		ATG	T			1	643	643		ATG	T	
tRNA-His(H)	644	705	62				GTG		644	707	64				GTG
*cytb*	707	1792	1086	1	ATG	TAA			708	1793	1086		ATG	TAA	
*nad*4L	1792	2052	261	-1	ATG	TAG			1793	2053	261	-1	ATG	TAA	
*nad*4	2013	3245	1233	-40	ATG	TAG			2014	3246	1233	-40	ATG	TAG	
tRNA-Gln(Q)	3247	3307	61	1			TTG		3247	3313	67				TTG
tRNA-Phe(F)	3304	3367	64	-4			GAA		3306	3369	64	-8			GAA
tRNA-Met(M)	3364	3425	62	-4			CAT		3364	3424	61	-6			CAT
*atp*6	3427	3942	516	1	ATG	TAA			3427	3942	516	2	ATG	TAG	
*nad*2	3943	4818	876		GTG	TAG			3942	4814	873	-1	GTG	TAG	
tRNA-Val (V)	4819	4879	61				TAC		4817	4876	60	2			TAC
tRNA-Ala (A)	4878	4938	61	-2			TGC		4875	4936	62	-2			TGC
tRNA-Asp(D)	4942	5002	61	3			GTC		4940	5002	63	3			GTC
*nad*1	5003	5896	894		ATG	TAG			5005	5898	894	2	ATG	TAG	
tRNA-Asn(N)	5896	5959	64	-1			GTT		5898	5960	63	-1			GTT
tRNA-Pro(P)	5962	6021	60	2			TGG		5963	6024	62	2			TGG
tRNA-Ile(I)	6021	6084	64	-1			GAT		6024	6087	64	-1			GAT
tRNA-Lys(K)	6085	6143	59				CTT		6088	6147	60				CTT
*nad*3	6144	6491	348		GTG	TAG			6148	6498	351		GTG	TAG	
tRNA-Ser(S1)	6489	6545	57	-3			GCT		6497	6552	56	-2			GCT
tRNA-Trp(W)	6547	6608	62	1			TCA		6555	6618	64	2			TCA
*cox*1	6613	8166	1554	4	ATG	TAG			6625	8166	1542	6	ATG	TAG	
tRNA-Thr(T)	8157	8217	61	-10			TGT		8157	8219	63	-10			TGT
16S	8218	9171	954						8220	9166	947				
tRNA-Cys(C)	9172	9230	59				GCA		9167	9230	64				GCA
12S	9231	9931	701						9231	9937	707				
tRNA-Leu(L1)	9932	9995	64				TAG		9938	10,002	65				TAG
tRNA-Ser(S2)	9998	10,059	62	2			TGA		10,009	10,072	64	6			TGA
tRNA-Leu(L2)	10,060	10,123	64				TAA		10,075	10,136	62	2			TAA
NCR1	10,124	10,996	873						10,137	10,344	208				
*cox*2	10,997	11,569	573		GTG	TAA			10,345	10,920	576		ATG	TAG	
tRNA-Glu(E)	11,570	11,640	71				TTC		10,921	10,987	67				TTC
*nad*6	11,641	12,099	459		GTG	TAG			10,988	11,446	459		GTG	TAG	
tRNA-Tyr(Y)	12,106	12,171	66	6			GTA		11,454	11,517	64	7			GTA
tRNA-Arg(R)	12,173	12,226	54	1			TCG		11,519	11,574	56	1			TCG
*nad*5	12,227	13,783	1557		GTG	TAG			11,577	13,124	1548	2	GTG	TAA	
tRNA-Gly(G)	13,784	13,847	64				TCC		13,124	13,186	63	-1			TCC
NCR2	13,848	15,130	1283						13,187	14,011	825				
(C) *Khawia sinensis*								(D) *Schyzocotyle acheilognathi* (CN)							
*cox*3	1	637	637		ATG	T			1	655	655		ATG	T	
tRNA-His(H)	638	699	62				GTG		656	730	75				GTG
*cytb*	701	1822	1122	1	ATG	TAA			734	1831	1098	3	ATG	TAG	
*nad*4L	1804	2064	261	-19	ATG	TAG			1833	2093	261	1	GTG	TAG	
*nad*4	2025	3257	1233	-40	ATG	TAA			2054	3304	1251	-40	GTG	TAG	
tRNA-Gln(Q)	3258	3318	61				TTG		3304	3367	64	-1			TTG
tRNA-Phe(F)	3315	3378	64	-4			GAA		3363	3426	64	-5			GAA
tRNA-Met(M)	3374	3436	63	-5			CAT		3423	3486	64	-4			CAT
*atp*6	3440	3955	516	3	ATG	TAA			3490	4005	516	3	ATG	TAG	
*nad*2	3960	4832	873	4	ATG	TAG			4006	4878	873		ATG	TAG	
tRNA-Val (V)	4835	4894	60	2			TAC		4883	4948	66	4			TAC
tRNA-Ala (A)	4893	4954	62	-2			TGC		4958	5019	62	9			TGC
tRNA-Asp(D)	4960	5024	65	5			GTC		5025	5087	63	5			GTC
*nad*1	5025	5918	894		ATG	TAG			5092	5985	894	4	ATG	TAA	
tRNA-Asn(N)	5918	5984	67	-1			GTT		5991	6055	65	5			GTT
tRNA-Pro(P)	5988	6047	60	3			TGG		6060	6122	63	4			TGG
tRNA-Ile(I)	6047	6110	64	-1			GAT		6128	6193	66	5			GAT
tRNA-Lys(K)	6117	6177	61	6			CTT		6198	6259	62	4			CTT
*nad*3	6178	6523	346		ATG	T			6264	6608	345	4	ATG	TAA	
tRNA-Ser(S1)	6524	6578	55				TCT		6607	6665	59	-2			GCT
tRNA-Trp(W)	6579	6641	63				TCA		6666	6729	64				TCA
*cox*1	6646	8196	1551	4	ATG	TAG			6742	8328	1587	12	ATG	TAG	
tRNA-Thr(T)	8187	8247	61	-10			TGT		8342	8404	63	13			TGT
								NCR1	8405	8528	124				
16S	8248	9193	946						8529	9494	966				
tRNA-Cys(C)	9194	9251	58				GCA		9495	9555	61				GCA
12S	9252	9960	709						9556	10,285	730				
tRNA-Leu(L1)	9961	10,023	63				TAG	*cox*2	10,286	10,858	573		ATG	TAA	
tRNA-Ser(S2)	10,025	10,087	63	1			TGA	E	10,862	10,924	63	3			TTC
tRNA-Leu(L2)	10,089	10,150	62	1			TAA	*nad*6	10,928	11,383	456	3	ATG	TAA	
NCR1	10,151	10,699	549					L1	11,402	11,465	64	18			TAG
*cox*2	10,700	11,273	574		ATG	T		L2	11,468	11,531	64	2			TAA
tRNA-Glu(E)	11,272	11,332	61	-2			TTC	Y	11,539	11,602	64	7			GTA
*nad*6	11,333	11,791	459		ATG	TAA		S2	11,620	11,685	66	17			TGA
tRNA-Tyr(Y)	11,797	11,859	63	5			GTA	NCR2	11,686	11,851	166				
tRNA-Arg(R)	11,872	11,925	54	12			TCG		11,852	11,909	58				TCG
*nad*5	11,926	13,476	1551		ATG	TAA			11,913	13,478	1566	3	ATG	TAA	
tRNA-Gly(G)	13,476	13,537	62	-1			TCC		13,484	13,547	64	5			TCC
NCR2	13,538	14,620	1083					NCR3	13,548	14,046	499				

### Phylogenetic analyses

Phylogenetic analysis was carried out using the mitogenomes generated from the four cestodes as part of this study as well as those of the 50 cestodes available from GenBank (Additional file 2: Table S2). Two trematodes, *Dicrocoelium chinensis* (NC_025279) and *Dicrocoelium dendriticum* (NC_025280), were used as outgroups. Another program written in-house, BioSuite [31], was employed to align all of the genes in batches using integrated MAFFT, wherein codon-alignment mode was used for the 12 PCGs, and normal alignment mode for the remaining genes (2 rRNAs and 22 tRNAs). The alignments were then concatenated to generate well-supported Phylip and nexus format files for use in the phylogenetic analysis software. Both the maximum likelihood (ML) and Bayesian inference (BI) were used to reconstruct phylogenetic trees, and selection of the most appropriate evolutionary models for the dataset was carried out using ModelGenerator v0.8527 [32]. Based on the Akaike information criterion, GTR + I + G was chosen as the optimal model for nucleotide evolution. ML analysis was performed by RaxML GUI [33] using an ML + rapid bootstrap algorithm with 1000 replicates. BI analysis was performed in MrBayes 3.2.1 [34] with default settings and 1 × 10^7^ Metropolis-coupled MCMC generations. The tree was then annotated using iTOL (a web-based tool) [35] with the help of several dataset files generated by MitoTool.

## Results

### Genome organisation and base composition

**Fig. 1:**
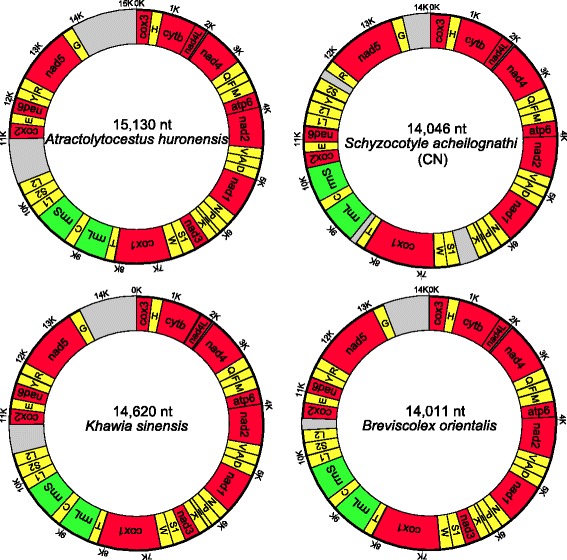
Map of the mitochondrial genomes of *Atractolytocestus huronensis*, *Breviscolex orientalis*, *Khawia sinensis* and *Schyzocotyle acheilognathi* (China, CN). The 12 protein-coding genes (PCGs), 22 tRNA and two rRNA genes are depicted as well as the non-coding regions (NCRs)

**Fig. 2:**
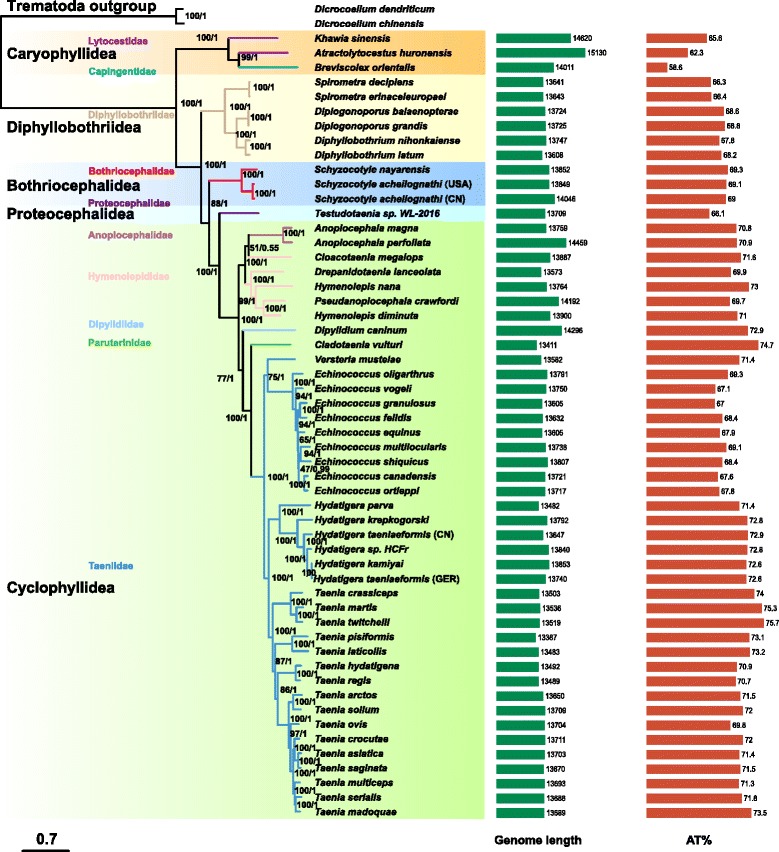
Maximum-likelihood tree inferred from 36 genes (12 protein-coding genes, 2 rRNAs and 22 tRNAs) of mitochondrial genomes of 54 cestode species from five orders, using two trematoda species as outgroups. Scale-bar represents the estimated number of substitutions per site. Bootstrap/posterior probability support values of ML/BI analysis are shown above the nodes. The bar graph (corresponding to tip labels in the tree) of the mitogenome length and A-T content are shown on the right of the tree

The mitogenomes of *A. huronensis* (GenBank accession number: KY486754), *B. orientalis* (KY486752), *K. sinensis* (KY486753) and *S. acheilognathi* (CN) (KX589243) are circular double-stranded DNA molecules. The size of these mitogenomes was 15,130 bp in *A. huronensis*, 14,620 bp in *K. sinensis*, 14,011 bp in *B. orientalis*, and 14,046 bp in *S. acheilognathi* (CN) (Fig. 1). The mitogenome of *A. huronensis* was the largest of all those available for cestodes (Additional file 2: Table S2, Fig. 2). The length of the *S. acheilognathi* (CN) mitogenome was about 140 bp longer than previously published due to the presence of a longer NCR between *nad*5 and *cox*3 [36]. Similar to other flatworm mitogenomes [11], which lacked the *atp8* gene, and encoded all the genes on the same strand, all of those generated in this study contained the standard 36 elements: 12 PCGs (*atp6*, *cytb*, *cox*1–3, *nad*1–6 and *nad4L*), 22 tRNA genes and two rRNA genes (Fig. 1). Intriguingly, A-T content of the three Caryophyllidea species (*K. sinensis*, *A. huronensis* and *B. orientalis*) was the lowest of all published cestode mitogenomes (Fig. 2).

### Protein-coding genes and codon usage

The size of the 12 PGCs ranged from 258 bp (*nad*4L) to 1554 bp (*nad*5) for the three caryophyllideans, but from 258 bp (*nad*4L) to 1584 bp (*cox*1) for *S. acheilognathi* (CN) (Additional file 3: Table S3). Only two types of start codons (ATG and GTG) were inferred from the sequence data of the four cestodes. GTG was used as a start codon for the following genes: *nad*2*, nad*3*, cox*2*, nad*5 and *nad*6 in *A. huronensis*, *nad*2*, nad*3*, nad*5 and *nad*6 in *B. orientalis* and *nad*4*, nad*4L in *S. acheilognathi* (CN). The rest of the PCGs of the aforementioned cestodes and all of the PCGs of *K. sinensis* used ATG as a start codon. From the three predicted stop codons, TAG, TAA and the abbreviated stop codon T, TAG was the most frequently occurring stop codon, followed by TAA and finally T. The unusual stop codon T encoded for *cox*3 in *A. huronensis*, *B. orientalis* and *S. acheilognathi* (CN) and *cox*2, *cox*3 and *nad*3 in *K. sinensis* (Table 1). RSCU for the four cestode mtDNAs calculated using the echinoderm mt genetic code are presented in Additional file 4: Figure S1. Overall, the three most commonly used T-rich codons for the three Caryophyllidea cestodes (*A. huronensis*, *B. orientalis* and *K. sinensis*) were Val (GTT), Leu (TTG) and Phe (TTT) compared with Tyr (TAT), Leu (TTG) and Phe (TTT) for *S. acheilognathi* (CN).

### Transfer and ribosomal RNA genes

All 22 tRNAs from the mt genome of each Caryophyllidea species were concatenated. This created a total concatenated length of 1363 bp, 1378 bp, 1354 bp and 1404 bp for *A. huronensis*, *B. orientalis*, *K. sinensis* and *S. acheilognathi* (CN), respectively (Additional file 3: Table S3). Each tRNA identified from these four species, could be folded into the traditional cloverleaf structure, with the exception of *tRNA* ^*Ser(AGN)*^ and *tRNA* ^*Arg*^ in *B. orientalis*, *K. sinensis* and *S. acheilognathi* (CN) and *tRNA* ^*Ser(AGN)*^, *tRNA* ^*Arg*^ and *tRNA* ^*Cys*^ in *A. huronensis*, which all lacked DHU arms (Additional file 5: Figure S2). All tRNAs had the standard anti-codons found in flatworms (Table 1), except *tRNA* ^*Ser(AGN)*^ in *K. sinensis* which had an anti-codon of TCT. The two ribosomal RNA genes, *rrnL* and *rrnS* were flanked by *tRNA* ^*Thr*^ and *cox*2 and separated by *tRNA* ^*Cys*^. This was identical in all the cestodes for which a mitogenome was available (Additional file 6: Figure S3). The boundary of the *rrnL* gene for *S. acheilognathi* (CN) was redefined, being approximately 100 bp shorter than that of previously published mitogenomes. This is due to the difference in defining the boundary (Additional file 7: Figure S4) [36]. Thus, there was an additional 124 bp NCR located between *tRNA* ^*Thr*^ and *rrnL*. Additionally, to conduct phylogenetic analysis and linear order comparison (see later), we proposed a reasonable *tRNA* ^*Gln*^ annotation to a recently reported mitogenome from *Testudotaenia* sp*. WL-2016* (KU761587) based upon alignments with other cestodes.

### Non-coding regions

**Fig. 3:**
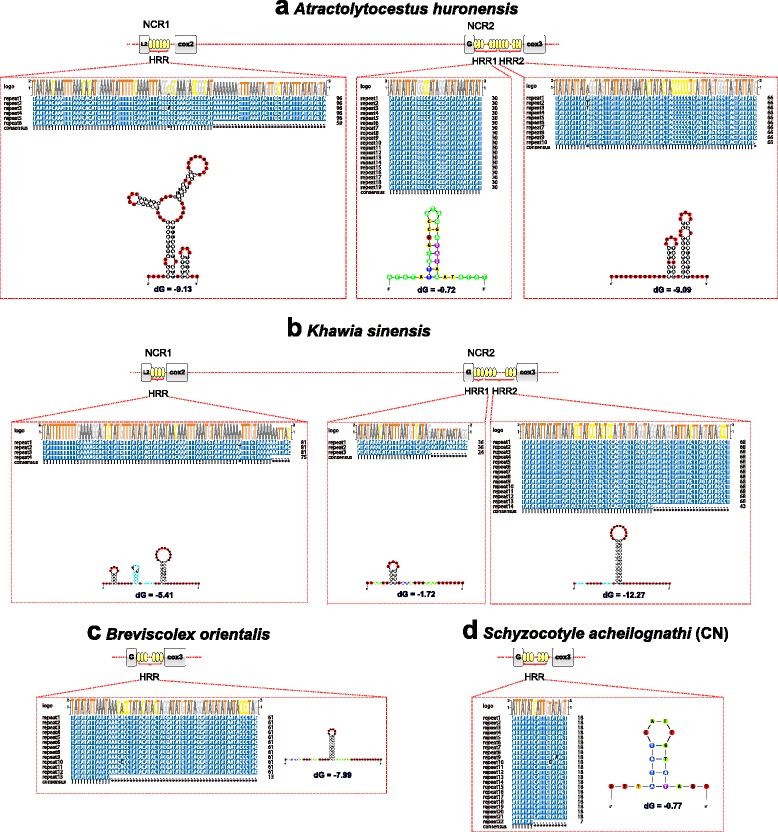
Highly repetitive regions (HRRs) and their secondary structures of the consensus repeat units in the major non-coding regions (NCRs) of the mitochondrial genomes of *Atractolytocestus huronensis* (**a**), *Khawia sinensis* (**b**), *Breviscolex orientalis* (**c**) and *Schyzocotyle acheilognathi* (China, CN) (**d**). Thermodynamic value (dG) is shown under the secondary structure

The position of the NCR in all cestodes was identified with a threshold value of 50 bp. The majority of cestodes contained two NCRs, except for *Pseudanoplocephala crawfordi* [37], *Taenia crocutae* [38], *Taenia solium* [39] and *S. acheilognathi* (CN) all of which had three NCRs, and *Hydatigera taeniaeformis* which has just one NCR. These NCRs occurred in the junctions of *rrnS*-*tRNA* ^*Arg*^ (P1) and *nad*5-*cox*3 (P2) (Additional file 6: Figure S3). The length of the major NCRs were 873 bp (NCR1) and 1283 bp (NCR2) in *A. huronensis*, 549 bp (NCR1) and 1083 bp (NCR2) in *K. sinensis*, 208 bp (NCR1) and 825 bp (NCR2) in *B. orientalis* and 124 bp (NCR1), 166 bp (NCR2) and 499 bp (NCR3) in *S. acheilognathi* (CN). The concatenated size (2156 bp) of all NCRs from *A. huronensis* was the longest of all the cestodes (Additional file 3: Table S3). Various highly repetitive regions (HRRs) were detected in NCRs from the four cestode species, and the consensus repeats were capable of forming stem loop structures (Fig. 3).

### Phylogeny and gene order

Both phylogenetic trees (BI and ML) demonstrated high statistical support for branch topology, especially on the order level (BP ≥ 85, BPP = 1). Since the two trees had the same topology, only the latter was shown (Fig. 2). The most derived Cyclophyllidea cestodes, together with the Proteocephalidea (represented by *Testudotaenia* sp. WL-2016), constitute a reciprocal monophyletic group with the Bothriocephalidea. This clade formed a sister-group to the Diphyllobothriidea, and all clades exhibited a sister-group relationship with the basal Caryophyllidea (Fig. 2). *Breviscolex orientalis* belonging to the family Capingentidae clustered into a well-supported clade with *A. huronensis* from the family Lytocestidae inferred by a maximum possible nodal support (BP = 100, BPP = 1) which formed a sister-group relationship with another Lytocestidae species, *K. sinensis*.

**Fig. 4:**
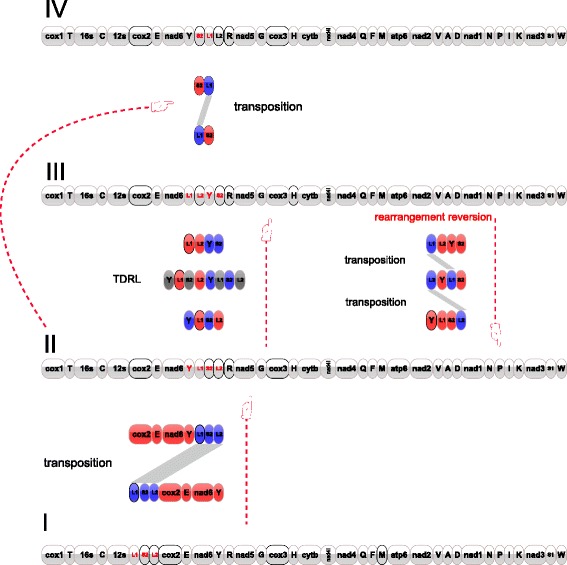
Rearrangement events predicted by CREx to explain gene order changes among the four mitogenome arrangements categories, Caryophyllidea (I), Diphyllobothriidea and Cyclophyllidea group 2 (II), Bothriocephalidea (III), Proteocephalidea and Cyclophyllidea group 1 (IV). L1, *tRNA* ^*Leu(CUN)*^; L2, *tRNA* ^*Leu(UUR)*^, S2, *tRNA* ^*Ser(UCN)*^; E, *tRNA* ^*Glu*^; Y, *tRNA* ^*Tyr*^; TDRL, tandem-duplication-random-loss

Amongst the 54 mitogenomes across the five orders, each order had a unique arrangement except for the Cyclophyllidea which had two types: group 1 (represented by the Taeniidae) was identical to the Diphyllobothriidea, and group 2 (represented by the Hymenolepididae, Anoplocephalidae, Dipylidiidae and Paruterinidae) was identical to the Proteocephalidea. These corresponded to four mt gene arrangement categories: I, Caryophyllidea; II, Diphyllobothriidea and group 1; III, Bothriocephalidea; IV, Proteocephalidea and group 2 (Fig. 4). Pairwise analysis between the four gene arrangement categories indicated similarities (common intervals algorithm) in the gene order between unsegmented and segmented cestodes to be lower than within segmented cestodes (Table 2).

**Table 2:** Pairwise comparisons of mitochondrial DNA gene orders among the four categories of mitogenome arrangements (see Fig. 4)

	I	II	III	IV
I	1254			
II	832	1254		
III	818	992	1254	
IV	828	1122	996	1254

Scores indicate the similarity between gene orders, where “1254” represents an identical gene order

## Discussion

In the phylogenetic analysis employed in this study, the Caryophyllidea was resolved as the sister taxon to all other eucestodes in line with previous studies. Although only five orders of cestodes are included in the phylogenetic analysis, the evolutionary relationships remain consistent with the results generated through morphological examination [1] and sequence data obtained from large fragments of mtDNA [4].

The mitogenome gene order of the cestodes was extremely conservative. Amongst the 54 mitogenomes across the five orders, only four gene arrangement categories were found. With respect to the three types of gene arrangements (II, III and IV) in the segmented cestodes, all the rearrangement operations are acted on the four closely linked tRNA genes (*tRNA* ^*Leu(CUN)*^-*tRNA* ^*Ser(UCN)*^-*tRNA* ^*Leu(UUR)*^-*tRNA* ^*Tyr*^) (Fig. 4). When compared with the category I in the unsegmented cestodes, there probably exists a long distance transposition event (the three tRNA genes *tRNA* ^*Leu(CUN)*^-*tRNA* ^*Ser(UCN)*^-*tRNA* ^*Leu(UUR)*^ translocate to the 3′ end of the four genes *cox*2-*tRNA* ^*Glu*^-*Nad6*-*tRNA* ^*Tyr*^) (Fig. 4), which may be the main cause of the low similarity value. According to the results of CREx program, the gene rearrangements from category II to category III and IV undergo a tandem-duplication-random-loss (TDRL) event and a simple transposition event, respectively. A TDRL event can provide directional information, allowing the inference of the ancestral state from the comparison of only two taxa because reversing the rearrangement would require more than a single operation [40]. Based on this assumption on TDRL event (Fig. 4), category II may be the ancestral state of the two categories II and III. Two categories of mt gene order were also found in the most derived Cyclophyllidea owing to the transposition of two tRNA genes [41]. However, the two types of gene arrangements are identical to those of the sister order Proteocephalidea and the relatively basal order Diphyllobothriidea.

There are perhaps more gene arrangements in other orders of cestodes; however, due to the limited amount of mitogenome data available so far, we can only but speculate. The rearrangement events that have been observed among the four arrangement categories in this study all took place in P1 as mentioned above (Fig. 4), revealing a rearrangement hot spot. Interestingly, P1 is furthermore the position in which one or two NCRs frequently occurred, and in which highly repetitive regions (HRRs) also are found within the NCRs. Whether an association exists between the rearrangement hot spot and the NCRs is something that requires further investigation to ascertain whether they may be important in the evolution of cestodes.

The phylogenetic relationship between *B. orientalis* and *A. huronensis* was found to be closer than that of *A. huronensis* and *K. sinensis*, which conflicts with classic systematics. On the basis of the paramuscular position of the vitelline follicles, *B. orientalis* is placed into the family Capingentidae Kulakovskaya, 1962, being the only member of this family found in the Palaearctic region. However, the fibres of the longitudinal musculature are situated mostly in the inner region of the vitelline field or entirely medullary to it, which is similar to the topography present in the Lytocestidae which possess cortically situated vitelline follicles [42]. *Breviscolex orientalis* has a cuneiform scolex, as do both species of *Caryophyllaeides* Nybelin, 1922 in the Lytocestidae [16]. These results suggest that the morphological characters of *B. orientalis* are closer to those of the Lytocestidae. Despite the similar result found in this study, relocation of *B. orientalis*, the only member of the family Capingentidae, into the family Lytocestidae, needs more molecular support.

## Conclusions

Among the four arrangement categories, the rearrangement events are detected in P1 where the NCRs with highly repetitive regions (HRRs) are common. A putative long-distance transposition event is detected between the unsegmented and segmented cestodes. The TDRL event suggests that the mt gene arrangement of the Diphyllobothriidea is the ancestral state relative to Bothriocephalidea. Gene arrangements of the Taeniidae and the rest of the families in the Cyclophyllidea are found to be identical to those of the sister order Proteocephalidea and the relatively basal order Diphyllobothriidea, respectively.

## Additional files

Additional file 1: Table S1. Primers used to amplify and sequence the mitochondrial genome of the cestodes *Atractolytocestus huronensis*, *Khawia sinensis*, *Breviscolex orientalis* and *Schyzocotyle acheilognathi* (CN). (XLSX 26 kb)

Additional file 2: Table S2. Characteristics of the 54 cestode mitochondrial genomes as well as two trematode outgroups in this study. (XLSX 20 kb)

Additional file 3: Table S3. Skewness and A + T content (%) of the protein-coding genes (PCGs), tRNAs, rRNA genes, each codon position of PCGs and non-coding region of the mitochondrial genome of the cestodes *Atractolytocestus huronensis*, *Khawia sinensis*, *Breviscolex orientalis* and *Schyzocotyle acheilognathi* (CN). (XLSX 16 kb)

Additional file 4: Figure S1. The relative synonymous codon usage (RSCU) values of the complete mitochondrial genome of the cestodes *Atractolytocestus huronensis*, *Khawia sinensis*, *Breviscolex orientalis* and *Schyzocotyle acheilognathi* (CN). (PDF 122 kb)

Additional file 5: Figure S2. Secondary structure (lacking DHU arms) of the tRNA genes of the cestodes *Atractolytocestus huronensis*, *Khawia sinensis*, *Breviscolex orientalis* and *Schyzocotyle acheilognathi* (CN). (PDF 472 kb)

Additional file 6: Figure S3. Mitochondrial gene order (include non-coding regions) of the 54 cestode species in this study. (PDF 3548 kb)

Additional file 7: Figure S4. The sequence alignment of the first 200 bp of the 16S rRNA gene from the 54 cestode species in this study. (PDF 635 kb)

## References

[1] Hoberg EP, Mariaux J, Justine JL, Brooks DR, Weekes PJ (1997). Phylogeny of the orders of the Eucestoda (Cercomeromorphae) based on comparative morphology: historical perspectives and a new working hypothesis. J Parasitol.

[2] Olson PD, Caira JN. Evolution of the major lineages of tapeworms (Platyhelminthes: Cestoidea) inferred from 18S ribosomal DNA and *elongation factor-1α*. J Parasitol. 1999;85(6):1134–59. 10647048

[3] Waeschenbach A, Webster BL, Bray RA, Littlewood DTJ (2007). Added resolution among ordinal level relationships of tapeworms (Platyhelminthes: Cestoda) with complete small and large subunit nuclear ribosomal RNA genes. Mol Phyogenet Evol..

[4] Waeschenbach A, Webster BL, Littlewood DTJ (2012). Adding resolution to ordinal level relationships of tapeworms (Platyhelminthes: Cestoda) with large fragments of mtDNA. Mol Phyogenet Evol..

[5] Ballard JWO, Whitlock MC (2004). The incomplete natural history of mitochondria. Mol Ecol.

[6] Huyse T, Buchmann K, Littlewood DTJ (2008). The mitochondrial genome of *Gyrodactylus derjavinoides* (Platyhelminthes: Monogenea) - a mitogenomic approach for *Gyrodactylus* species and strain identification. Gene.

[7] Zarowiecki MZ, Huyse T, Littlewood DTJ (2007). Making the most of mitochondrial genomes - markers for phylogeny, molecular ecology and barcodes in *Schistosoma* (Platyhelminthes: Digenea). Int J Parasitol.

[8] Boore JL, Brown WM (1998). Big trees from little genomes: mitochondrial gene order as a phylogenetic tool. Curr Opin Genet Dev.

[9] Boore J (2006). The use of genome-level characters for phylogenetic reconstruction. Trends Ecol Evol.

[10] Masta SE, McCall A, Longhorn SJ (2010). Rare genomic changes and mitochondrial sequences provide independent support for congruent relationships among the sea spiders (Arthropoda, Pycnogonida). Mol Phyogenet Evol..

[11] Le TH, Blair D, McManus DP (2002). Mitochondrial genomes of parasitic flatworms. Trends Parasitol.

[12] Littlewood DTJ, Lockyer AE, Webster BL, Johnston DA, Le TH (2006). The complete mitochondrial genomes of *Schistosoma haematobium* and *Schistosoma spindale* and the evolutionary history of mitochondrial genome changes among parasitic flatworms. Mol Phyogenet Evol..

[13] Park JK, Kim KH, Kang S, Kim W, Eom KS, Littlewood DTJ. A common origin of complex life cycles in parasitic flatworms: evidence from the complete mitochondrial genome of *Microcotyle sebastis* (Monogenea: Platyhelminthes). BMC Evol Biol. 2007;7:11. 10.1186/1471-2148-7-11 PMC1800851 17270057

[14] Oros M, Hanzelova V, Scholz T (2004). The cestode *Atractolytocestus huronensis* (Caryophyllidea) continues to spread in Europe: new data on the helminth parasite of the common carp. Dis Aquat Org.

[15] Oros M, Hanzelová V, Scholz T (2009). Tapeworm *Khawia sinensis*: review of the introduction and subsequent decline of a pathogen of carp, *Cyprinus carpio*. Vet Parasitol.

[16] Oros M, Scholz T, Hanzelová V, Mackiewicz JS (2010). Scolex morphology of monozoic cestodes (Caryophyllidea) from the Palaearctic region: a useful tool for species identification. Folia Parasitol.

[17] Králová-Hromadová I, Štefka J, Špakulová M, Orosová M, Bombarová M, Hanzelová V (2010). Intra-individual internal transcribed spacer 1 (ITS1) and ITS2 ribosomal sequence variation linked with multiple rDNA loci: a case of triploid *Atractolytocestus huronensis*, the monozoic cestode of common carp. Int J Parasitol.

[18] Littlewood D, Waeschenbach A, Nikolov P (2008). In search of mitochondrial markers for resolving the phylogeny of cyclophyllidean tapeworms (Platyhelminthes, Cestoda) - a test study with Davaineidae. Acta Parasitol.

[19] Katoh K, Standley DM (2013). MAFFT multiple sequence alignment software version 7: improvements in performance and usability. Mol Biol Evol.

[20] Altschul SF, Gish W, Miller W, Myers EW, Lipman DJ (1990). Basic local alignment search tool. J Mol Biol.

[21] Lowe TM, Eddy SR (1997). tRNAscan-SE: a program for improved detection of transfer RNA genes in genomic sequence. Nucleic Acids Res.

[22] Laslett D, Canback B (2008). ARWEN: a program to detect tRNA genes in metazoan mitochondrial nucleotide sequences. Bioinformatics.

[23] Bernt M, Donath A, Juhling F, Externbrink F, Florentz C, Fritzsch G (2013). MITOS: improved *de novo* metazoan mitochondrial genome annotation. Mol Phyogenet Evol..

[24] Wyman SK, Jansen RK, Boore JL (2004). Automatic annotation of organellar genomes with DOGMA. Bioinformatics.

[25] Zhang D (2016). MitoTool software.

[26] Benson G (1999). Tandem repeats finder: a program to analyze DNA sequences. Nucleic Acids Res.

[27] Beitz E (2000). T(E)Xshade: shading and labeling of multiple sequence alignments using (LTEX)-T-A 2(epsilon). Bioinformatics.

[28] Zuker M (2003). Mfold web server for nucleic acid folding and hybridization prediction. Nucleic Acids Res.

[29] Tamura K, Peterson D, Peterson N, Stecher G, Nei M, Kumar S (2011). MEGA5: molecular evolutionary genetics analysis using maximum likelihood, evolutionary distance, and maximum parsimony methods. Mol Biol Evol.

[30] Bernt M, Merkle D, Ramsch K, Fritzsch G, Perseke M, Bernhard D (2007). CREx: inferring genomic rearrangements based on common intervals. Bioinformatics.

[31] Zhang D (2016). BioSuite software.

[32] Keane TM, Creevey CJ, Pentony MM, Naughton TJ, McLnerney JO (2006). Assessment of methods for amino acid matrix selection and their use on empirical data shows that ad hoc assumptions for choice of matrix are not justified. BMC Evol Biol.

[33] Silvestro D, Michalak I (2011). raxmlGUI: a graphical front-end for RAxML. Org Divers Evol.

[34] Ronquist F, Teslenko M, van der Mark P, Ayres DL, Darling A, Höhna S (2012). MrBayes 3.2: efficient Bayesian phylogenetic inference and model choice across a large model space. Syst Biol.

[35] Letunic I, Bork P (2016). Interactive tree of life (iTOL) v3: an online tool for the display and annotation of phylogenetic and other trees. Nucleic Acids Res.

[36] Brabec J, Kuchta R, Scholz T, Littlewood DTJ (2016). Paralogues of nuclear ribosomal genes conceal phylogenetic signals within the invasive Asian fish tapeworm lineage: evidence from next generation sequencing data. Int J Parasitol.

[37] Zhao GH, Wang HB, Jia YQ, Zhao W, Hu XF, Yu SK, et al. The complete mitochondrial genome of *Pseudanoplocephala crawfordi* and a comparison with closely related cestode species. J Helminthol. 2016;90(5):588–95. 10.1017/S0022149X15000802 26376709

[38] Terefe Y, Hailemariam Z, Menkir S, Nakao M, Lavikainen A, Haukisalmi V (2014). Phylogenetic characterisation of *Taenia* tapeworms in spotted hyenas and reconsideration of the “out of Africa” hypothesis of *Taenia* in humans. Int J Parasitol.

[39] Nakao M, Sako Y, Ito A (2003). The mitochondrial genome of the tapeworm *Taenia solium*: a finding of the abbreviated stop codon U. J Parasitol.

[40] Perseke M, Fritzsch G, Ramsch K, Bernt M, Merkle D, Middendorf M (2008). Evolution of mitochondrial gene orders in echinoderms. Mol Phyogenet Evol.

[41] Guo A (2016). The complete mitochondrial genome of the tapeworm *Cladotaenia vulturi* (Cestoda: Paruterinidae): gene arrangement and phylogenetic relationships with other cestodes. Parasit Vectors.

[42] Scholz T, Shimazu T, Olson PD, Nagasawa K (2001). Caryophyllidean tapeworms (Platyhelminthes: Eucestoda) from freshwater fishes in Japan. Folia Parasitol.

