# Supplementary material for: The complete mitochondrial DNA of three monozoic tapeworms in the Caryophyllidea: a mitogenomic perspective on the phylogeny of eucestodes
Source: Parasit Vectors. 2017 Jun 27;10:314. doi: 10.1186/s13071-017-2245-y (PMC5488446; doi:10.1186/s13071-017-2245-y)
Supplement: Supplementary file 4 — The relative synonymous codon usage (RSCU) values of the complete mitochondrial genome of the cestodes Atractolytocestus huronensis, Khawia sinensis, Breviscolex orientalis and Schyzocotyle acheilognathi (CN). (PDF 122 kb) [file 13071_2017_2245_MOESM4_ESM.pdf]

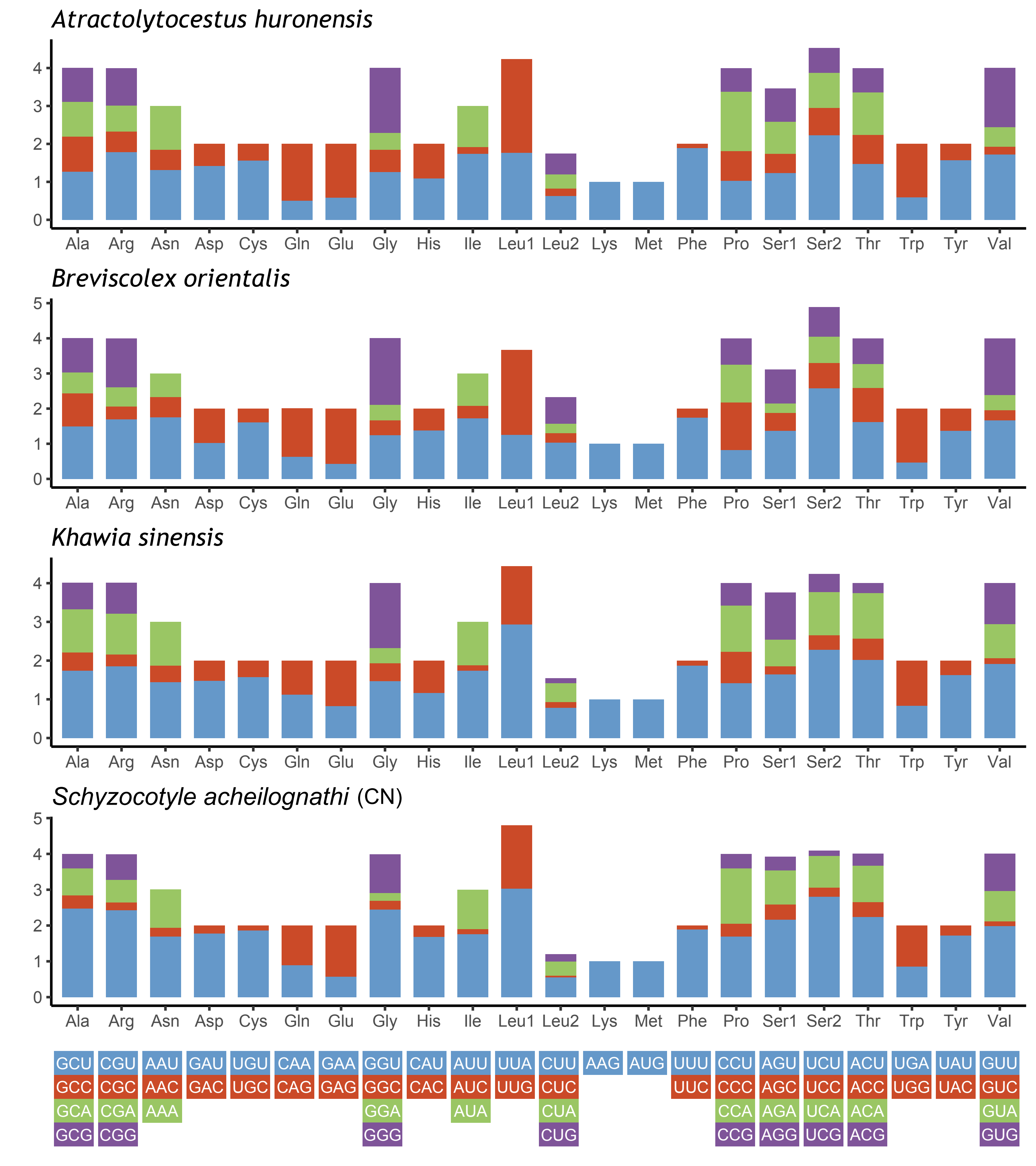

Atractolytocestus huronensis
Breviscolex orientalis
Khawia sinensis
Schyzocotyle acheilognathi (CN)
0
1
2
3
4
5
Ala
Arg
Asn
Asp
Cys
Gln
Glu
Gly
His
Ile
Leu1
Leu2
Lys
Met
Phe
Pro
Ser1
Ser2
Thr
Trp
Tyr
Val
GCU CGU AAU GAU UGU CAA GAA GGU CAU AUU UUA CUU AAG AUG UUU CCU AGU UCU ACU UGA UAU GUU
GCC CGC AAC GAC UGC CAG GAG GGC CAC AUC UUG CUC UUC CCC AGC UCC ACC UGG UAC GUC
GCA CGA AAA GGA AUA CUA CCA AGA UCA ACA GUA
GCG CGG GGG CUG CCG AGG UCG ACG GUG
