# Supplementary material for: The complete mitochondrial DNA of three monozoic tapeworms in the Caryophyllidea: a mitogenomic perspective on the phylogeny of eucestodes
Source: Parasit Vectors. 2017 Jun 27;10:314. doi: 10.1186/s13071-017-2245-y (PMC5488446; doi:10.1186/s13071-017-2245-y)
Supplement: Supplementary file 5 — Secondary structure (lacking DHU arms) of the tRNA genes of the cestodes Atractolytocestus huronensis, Khawia sinensis, Breviscolex orientalis and Schyzocotyle acheilognathi (CN). (PDF 472 kb) [file 13071_2017_2245_MOESM5_ESM.pdf]

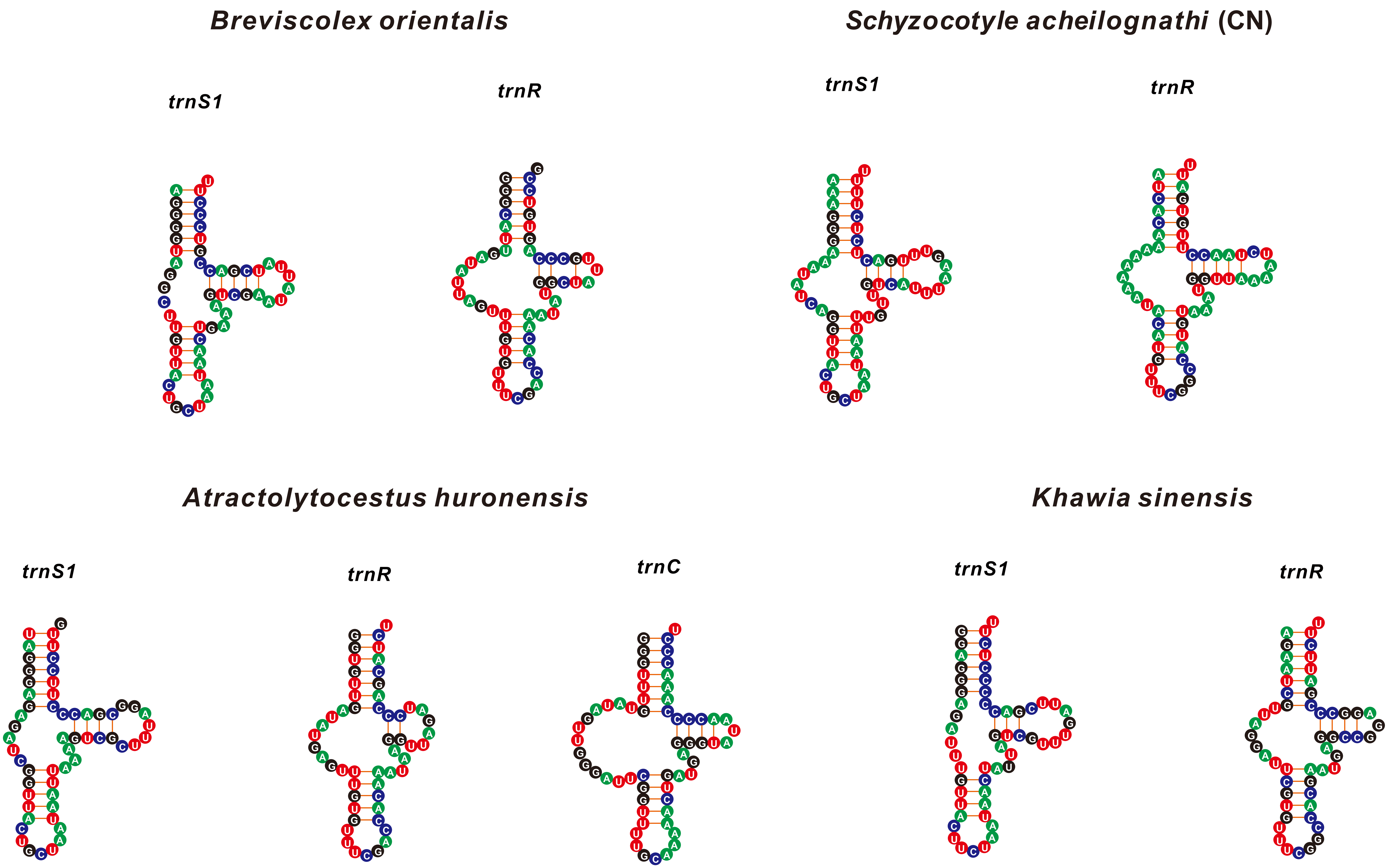

Breviscolex orientalis
trnS1
trnR
Schyzocotyle acheilognathi (CN)
trnS1
trnR
Atractolytocestus huronensis
trnS1
trnR
trnC
Khawia sinensis
trnS1
trnR
