# Supplementary material for: The complete mitochondrial DNA of three monozoic tapeworms in the Caryophyllidea: a mitogenomic perspective on the phylogeny of eucestodes
Source: Parasit Vectors. 2017 Jun 27;10:314. doi: 10.1186/s13071-017-2245-y (PMC5488446; doi:10.1186/s13071-017-2245-y)
Supplement: Supplementary file 6 — Mitochondrial gene order (include non-coding regions) of the 54 cestode species in this study. (PDF 3548 kb) [file 13071_2017_2245_MOESM6_ESM.pdf]

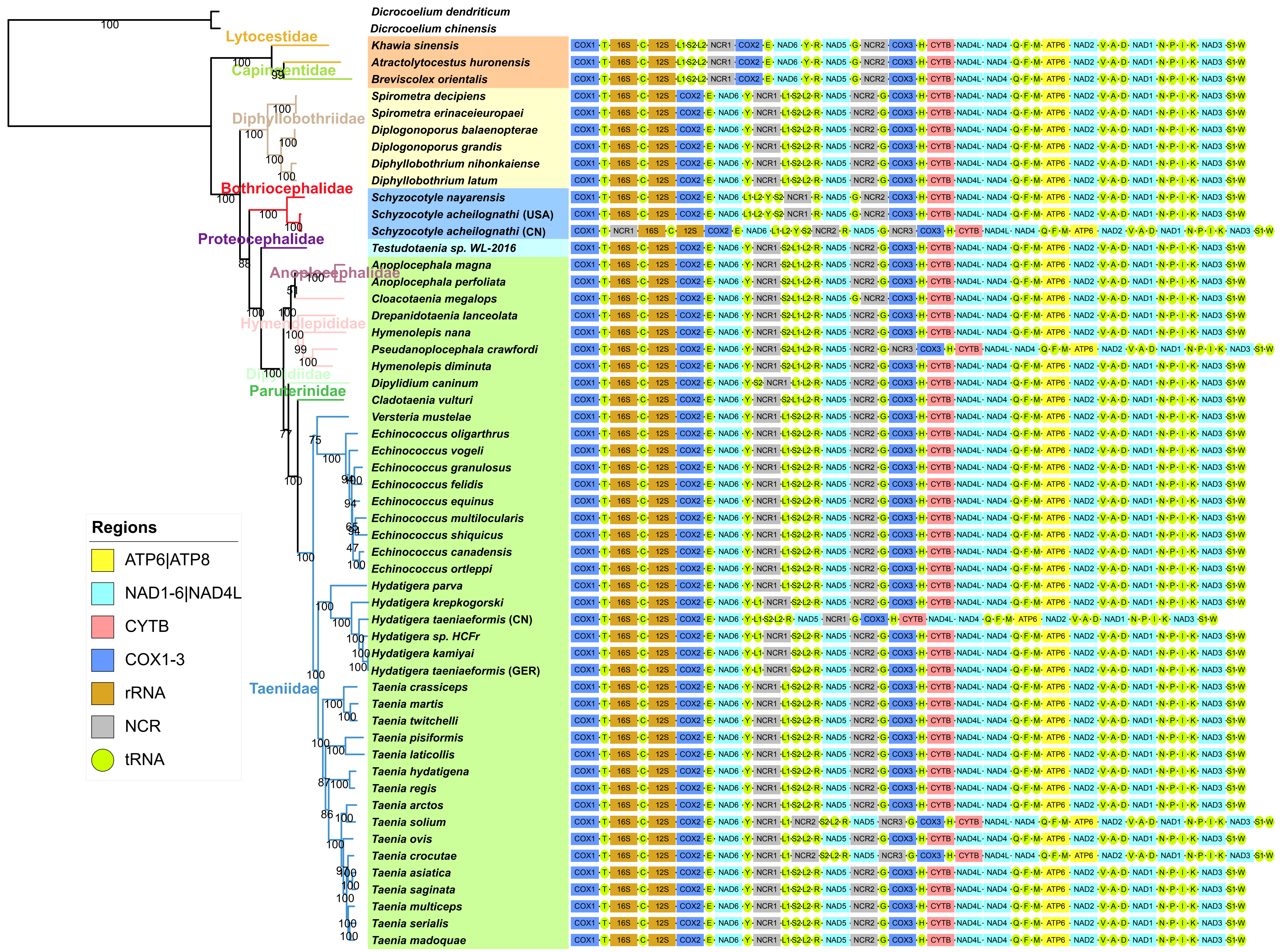

Dicrocoelium dendriticum
Dicrocoelium chinensis
Lytocestidae
Khawia sinensis
Atractolytocestus huronensis
Breviscolex orientalis
Capingentidae
Diphyllobothriidae
Spirometra decipiens
Spirometra erinaceieuropaei
Diplogonoporus balaenopterae
Diplogonoporus grandis
Diphyllobothrium nihonkaiense
Diphyllobothrium latum
Bothriocephalidae
Schyzocotyle nayarensis
Schyzocotyle acheilognathi (USA)
Schyzocotyle acheilognathi (CN)
Proteocephalidae
Testudotaenia sp. WL-2016
Anoplocephalidae
Anoplocephala magna
Anoplocephala perfoliata
Cloacotaenia megalops
Drepanidotaenia lanceolata
Hymenolepididae
Hymenolepis nana
Pseudanoplocephala crawfordi
Hymenolepis diminuta
Dipylidiidae
Dipylidium caninum
Paruterinidae
Cladotaenia vulturi
Versteria mustelae
Echinococcus oligarthrus
Echinococcus vogeli
Echinococcus granulosus
Echinococcus felidis
Echinococcus equinus
Echinococcus multilocularis
Echinococcus shiquicus
Echinococcus canadensis
Echinococcus ortleppi
Hydatigera parva
Hydatigera krepkogorski
Hydatigera taeniaeformis (CN)
Hydatigera sp. HCFr
Hydatigera kamiyai
Hydatigera taeniaeformis (GER)
Taeniidae
Taenia crassiceps
Taenia martis
Taenia twitchelli
Taenia pisiformis
Taenia laticollis
Taenia hydatigena
Taenia regis
Taenia arctos
Taenia solium
Taenia ovis
Taenia crocutae
Taenia asiatica
Taenia saginata
Taenia multiceps
Taenia serialis
Taenia madoquae
Regions
ATP6|ATP8
NAD1-6|NAD4L
CYTB
COX1-3
rRNA
NCR
tRNA
