# Supplementary figures and images for: The complete mitochondrial DNA of three monozoic tapeworms in the Caryophyllidea: a mitogenomic perspective on the phylogeny of eucestodes
Source: Parasit Vectors. 2017 Jun 27;10:314. doi: 10.1186/s13071-017-2245-y (PMC5488446; doi:10.1186/s13071-017-2245-y)

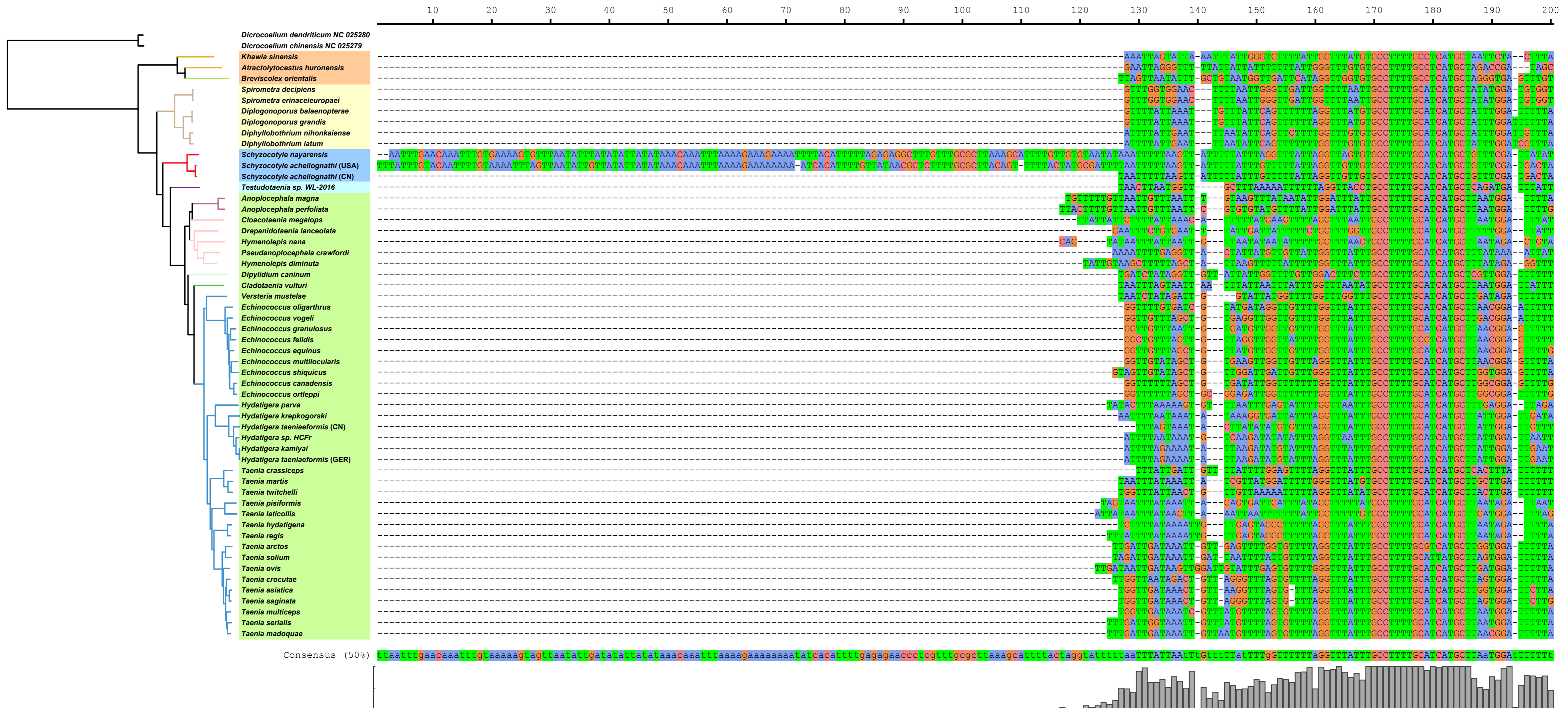

Supplement: Supplementary file 7 — The sequence alignment of the first 200 bp of the 16S rRNA gene from the 54 cestode species in this study. (PDF 635 kb) [file 13071_2017_2245_MOESM7_ESM.pdf]
